# Home invasion of triatomines (Hemiptera: Reduviidae) in the urban area of Mato Grosso, Brazil

**DOI:** 10.1590/0037-8682-0119-2025

**Published:** 2025-09-22

**Authors:** Janaina Cipriana dos Santos, Mirian Francisca Martins, Ludier Kesser Santos-Silva

**Affiliations:** 1 Universidade Federal de Mato Grosso, Programa de Pós-Graduação Stricto Sensu em Imunologia e Parasitologia, Barra do Garças, MT, Brasil.; 2 Escritório Regional de Saúde/Secretaria de Estado de Saúde, Departamento de Vigilância em Saúde Ambiental, Barra do Garças, MT, Brasil.; 3 Universidade Federal de Mato Grosso, Instituto de Ciências Biológicas e da Saúde, Laboratório de Análises Cromossômicas e Moleculares, Pontal do Araguaia, MT, Brasil.

**Keywords:** Triatominae, Food sources, Chagas disease, Entomological surveillance, Trypanosoma cruzi

## Abstract

**Background:**

Chagas disease, an anthropozoonosis endemic to Latin America, is caused by *Trypanosoma cruzi* and is a serious public health concern.

**Methods:**

We investigated the natural infection of triatomine bugs, genotyped *T. cruzi*, and identified the blood meal sources of the infected vectors in the Medio Araguaia region of Mato Grosso, Brazil.

**Results:**

In total, 235 triatomines were identified. The highest triatomine occurrence (95.7%) was observed in the municipality of Barra do Garças. The most prevalent species was *Triatoma williami* (89.7%), followed by *Rhodnius neglectus* (8.8%), *Panstrongylus geniculatus* (0.88%), and *Panstrongylus diasi* (0.44%). Barra do Garças showed a high rate of natural infection by *T. cruzi* (65.7%). Four discrete typing units were identified in the infected insects: TcIV and TcII strains in *T. williami*, and TcI and TcIII associated with *R. neglectus* and *P. geniculatus*, respectively. Regarding blood meal sources, *T. williami*, *P. geniculatus*, and *R. neglectus* predominantly fed on birds and rodents. However, human blood was detected in 32.8% of the insects.

**Conclusion:**

Overall, these findings indicate a high risk of Chagas disease vector transmission in the municipality of Barra do Garças, highlighting the need for innovative approaches to control and prevent this disease.

## INTRODUCTION

Chagas disease (ChD) is an infectious disease that affects seven million people worldwide, mostly in Latin America^1^. This disease has economic and social impacts^2^ ^-^ ^4^. ChD is classified as a neglected tropical disease and is among the parasitic diseases with the highest mortality rates worldwide. The scenario of this disease in Brazil is even more concerning, with an estimated 1.2-4.6 million people infected^1^ ^,^ ^4^ ^-^ ^6^.

The causative agent of ChD is *Trypanosoma cruzi* (Chagas, 1909) (Euglenozoa: Kinetoplastea: Trypanosomatidae), a hemoflagellate protozoan that has been categorized into seven lineages: TcI-TcVI and TcBat. These lineages are referred to as discrete typing units (DTUs) and differ in their genotypes, pathogenic mechanisms, and ecoepidemiological characteristics^7^ ^-^ ^9^. *T. cruzi* can be transmitted via several routes, including triatomine vectors (Hemiptera: Reduviidae: Triatominae). These vectors play a crucial role in the parasite transmission cycle^10^ ^-^ ^13^. Another factor contributing to *T. cruzi* transmission is the presence of mammals infected by the parasite^14^ ^-^ ^16^. These parasitized mammals serve as reservoirs encompassing various wild and domestic mammals. Dogs, cats, and pigs are the primary reservoirs of *T. cruzi* in domestic environments, while opossums, bats, and rodents are synanthropic reservoirs in the wild environment^15^ ^,^ ^17^.

Triatomines are blood-sucking insects popularly known as kissing bugs (*barbeiros* in Portuguese). A total of 64 species of kissing bugs have been described in Brazil, which are widely distributed across the country and have vector potential for *T. cruzi* transmission^18^. One Brazilian state with the greatest triatomine diversity is Mato Grosso (MT), where *Triatoma sordida* (Stål, 1859) and *Rhodnius neglectus* (Lent, 1954) are the most reported species^19^. Triatomine species are frequently reported to invade households^20^ ^,^ ^21^.

Surveillance of synanthropic triatomine vectors is necessary to determine the rate of natural infection (NI) by *T. cruzi* and to analyze the spatial distribution of vectors, thereby detecting the most vulnerable areas. This enables the assessment of vector relationships between domestic and wild cycles, colonization, and infestation rates, as well as the presence of domiciled vector species and, in certain cases, to identify circulating *T. cruzi* strains. This information helps to identify areas at the greatest risk of disease transmission and supports the implementation of vector control actions^22^ ^,^ ^23^.

The occurrence of infected triatomines in domestic environments has already been described in MT^24^ ^-^ ^28^, indicating the risk of ChD vector transmission. Considering the need to improve ChD health surveillance in this region and the limited number of published studies on the entomological monitoring of triatomines in the MT, this study aimed to perform an entomological analysis of ChD vectors to determine NI rates and identify *T. cruzi* reservoirs in the Medio Araguaia region. 

## MATERIAL AND METHODS

### Study area

**FIGURE 1:**
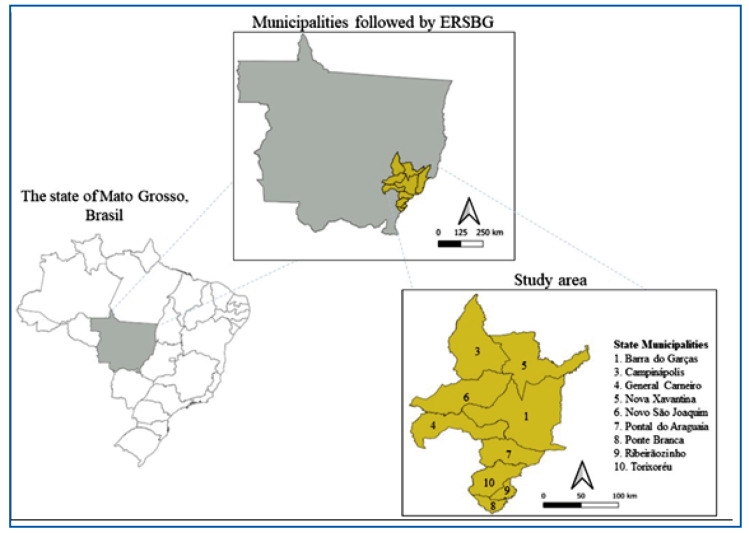
Map showing the state of Mato Grosso and the studied municipalities.

The Medio Araguaia region is located east of MT. Information regarding the entomological surveillance of ChD in this study was obtained from the *Escritório Regional de Saúde de Barra do Garças* (ERSBG). This Environmental Health Surveillance Agency covers the nine municipalities investigated: Barra do Garças, Campinápolis, General Carneiro, Nova Xavantina, Novo São Joaquim, Pontal do Araguaia, Ponte Branca, Ribeirãozinho, and Torixoréu (Figure 1 **,** Supplementary Table 1).

### Collection, processing, and parasitological examination of the sampled triatomines

Triatomines were collected through passive searches in urban and rural areas. These insects were sent to the entomology laboratory at the ERSBG for taxonomic identification using keys^29^ ^,^ ^30^. For each triatomine specimen, data regarding the capture site (intra- or peridomiciliary), sex, developmental stage, and preservation status (fresh or dry) were recorded. Parasitological examinations were conducted on fresh specimens to determine the NI rate of *T. cruzi*. This examination was performed on intestinal contents extracted for subsequent analysis. Fecal samples from 157 triatomines were stained with 10% Giemsa for microscopic examination^31^. Dry specimens (n = 78) were excluded from the parasitological and molecular analyses.

### Triatomine DNA extraction and DNA quality assessment

DNA from insect intestinal content samples was extracted using the Promega Wizard® SV Genomic DNA Purification System Kit (Promega, Madison, WI, USA) according to the manufacturer’s instructions. The concentration and purity of DNA samples were determined using a NanoDrop spectrophotometer (Thermo Scientific, Waltham, MA, USA). Uninfected *R. neglectus* maintained under laboratory conditions were used as negative controls for DNA extraction to prevent potential cross-contamination. Before DNA extraction and PCR, all work surfaces and instruments were decontaminated with a sodium hypochlorite solution, followed by exposure to UV light to minimize contamination and ensure accuracy of the results. Furthermore, all DNA extractions and PCR were conducted in separate laboratory rooms where no post-PCR procedures were conducted^32^. Next, conventional polymerase chain reaction (cPCR) was performed using primers P2bF/P6R, which amplify a 163 bp region of the triatomine 12S ribosomal RNA (rRNA) gene^33^. This PCR was used to analyze the quality of the extracted DNA samples. Samples exhibiting PCR amplification inhibition were excluded from subsequent analyses.

### Natural infection rates and genotyping of T. cruzi using conventional PCR

P21 forward and reverse primers, which amplify a 65 bp region of the T. cruzi genomic single-copy P21 gene, were used to evaluate the NI rates of *T. cruzi*. The P21 gene is species-specific and shows better specificity and optimization in PCR assays than the kinetoplast DNA. The PCR conditions were adapted from a previously described protocol^34^. A positive control (reaction containing *T. cruzi* DNA from the Colombian strain, TcI), a negative control (*R. neglectus* DNA), and a non-template control were used for cPCR (Supplementary Table 2). *T. cruzi*-positive samples were genotyped to determine DTUs.

Samples for genotyping were classified according to the triatomine species and the neighborhood where the insects were collected. First, samples from triatomines infected with *T. cruzi* were subjected to cPCR using primers that amplify *TcSC5D* gene^35^ (Supplementary Table 2). The amplified products were sequenced using Sanger sequencing. Electropherograms were obtained from the nucleotide sequences, which were analyzed using Chromas Lite^36^ and Geneious 4.8.3 programs. The forward and reverse sequences obtained from each sample were aligned with the sequence of the *TcSC5D* region of *T. cruzi* X10 Sylvio using Clustal Omega. The nucleotides of polymorphic sites 138, 168, 336, 495, 618, and 747 were compared to differentiate *T. cruzi* strains^35^.

### Triatomine food sources

DNA in the intestinal contents of insects has been evaluated for the presence of dogs, rodents, birds, and humans DNAs using the same samples as those used for genotyping. Insects were randomly selected from 50% of the household units (HU) to determine the presence of DNA from dogs, birds, and rodents (Supplementary Table 3). Therefore, cPCR was performed for 41 samples^37^ ^,^ ^38^ (Supplementary Table 2). DNA samples from both *T. cruzi*-infected and non-infected triatomines were analyzed to determine that human blood served as the food source.

### Entomological indicators

The total number of sampled triatomines, the number of triatomines found in domestic and peridomestic environments, (Number of house holds with nymphs)/(Total number of house holds with triatomines)×100), and natural infection index (Number of infected triatomines/Number of triatomines examined×100) were the entomological indicators evaluated^39^.

### Map preparation

The geographic coordinates of the HUs with positive triatomine detection in Barra do Garças were obtained using Garmin Etrex 10 GPS. Information on the neighborhoods of Barra do Garças was obtained from the municipality’s website (https://www.barradogarcas.mt.leg.br). The triatomine distribution was visualized via density maps (heat maps) using a Kernel density estimation algorithm prepared in QGIS® (version 3.10). 

### Statistical analysis

Statistical tests were performed using R Studio (version 4.0.2) with a significance level of 5%. Chi-square tests were used to assess the association between the NI rates of *R. neglectus* and *Triatoma williami*. Additionally, we evaluated the feeding preferences of *T. williami* and investigated whether the presence of human DNA in these triatomines was associated with their sex and the environment in which they were sampled. Finally, we evaluated whether triatomine invasion in the HUs of the municipality of Barra do Garças, MT, was influenced by proximity to the Parque Estadual da Serra Azul (PESA) ecological park using the Kendall correlation test.

## RESULTS

### Triatominae fauna and spatial distribution

**FIGURE 2:**
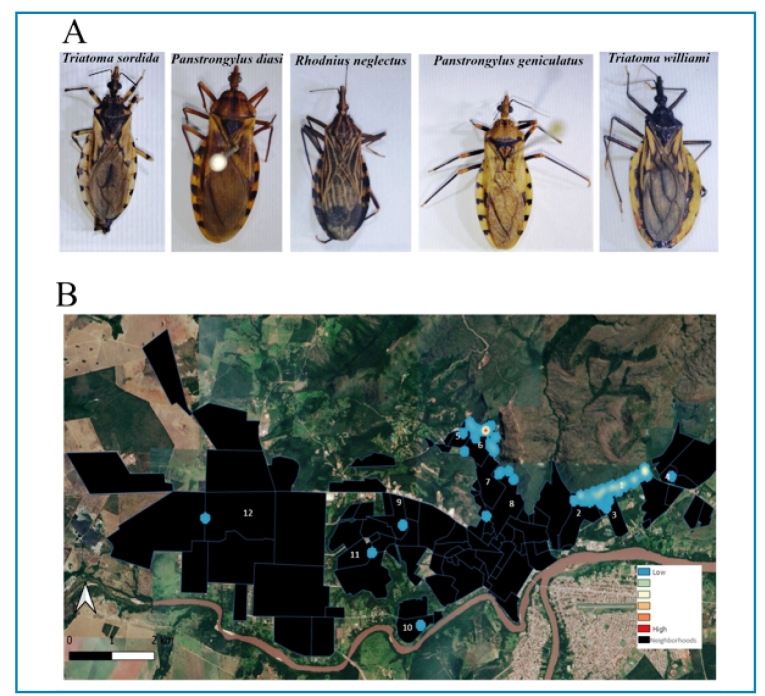
Triatomine species collected and their distribution. **A.** Triatomine species captured in the study area. **B.** Heat map showing the triatomine distribution by neighborhoods in Barra do Garças, Mato Grosso, between the years 2017 and 2019: 1) Jardim Amazônia I; 2) Jardim Mariano; 3) Jardim Paraíso; 4) Serra Azul Condominium; 5) Jardim Araguaia; 6) Jardim Pitaluga; 7) Vila Serrinha; 8) Vila Santo Antônio; 9) Jardim Piracema; 10) Jardim Toledo; 11) Wilmar Peres; 12) São José. PESA: Parque Estadual da Serra Azul Ecological Park. The heat gradient represents areas with the highest density of vectors.

Between 2017 and 2019, 235 triatomines were collected from Barra do Garças, Campinápolis, Nova Xavantina, Novo São Joaquim, Pontal do Araguaia, and Ribeirãozinho (Table 1). Barra do Garças was the municipality with the highest triatomine occurrence, accounting for 95.7% (225 insects) of the collected insects, with a predominance of the species *T. williami* (89.7%; 202 insects) (Table 1 **,** Figure 2A). Furthermore, *R. neglectus* (8.8%; 20 insects), *Panstrongylus geniculatus* (Latreille, 1811) (0.88%; two insects), and *Panstrongylus diasi* (Pinto & Lent, 1946) (0.44%; one insect) were found in this municipality (Table 1 **,** Figure 2A).

Pontal do Araguaia, a municipality surrounding Barra do Garças, had five insects collected, including *P. geniculatus* and *P. diasi*. *T. sordida* was found in Nova Xavantina, Novo São Joaquim, and Ribeirãozinho (Figure 2A). *R. neglectus* was found in Campinápolis (Table 1). Adult insects were observed in these municipalities, whereas nymphs were only found in Barra do Garças and Nova Xavantina. The nymphs captured in Barra do Garças were primarily collected from a single household with adult insects (males and females) and eggs. All insects were *T. williami* and formed a colony within the household, as previously described^28^.

**TABLE 1:** Number of triatomines sampled annually, species, and stage of development captured in six municipalities in the Middle Araguaia region.

Year	Municipality	Species	Developmental stage								Total
			N1^A^	N2^A^	N3^A^	N4^A^	N5^A^	^B^ ♂	^C^ **♀**	NI^D^	
2017	Barra do Garças	*Triatoma williami*						10	28	1	39
	Barra do Garças	*Rhodnius neglectus*							2		2
	Pontal do Araguaia	*Panstrongylus geniculatus*							1		1
		**Total**						**10**	**31**	**1**	**42**
2018	Barra do Garças	*Triatoma williami*						3	19	4	26
	Barra do Garças	*Rhodnius neglectus*						3			3
	Novo São Joaquim	*Triatoma sordida*						1			1
		**Total**						**7**	**19**	**4**	**30**
2019	Barra do Garças	*Triatoma williami*	41	1			1	23	71		137
	Barra do Garças	*Rhodnius neglectus*						9	5	1	15
	Barra do Garças	*Panstrongylus geniculatus*						2			2
	Barra do Garças	*Panstrongylus diasi*							1		1
	Campinápolis	*Rhodnius neglectus*						1			1
	Nova Xavantina	*Triatoma sordida*					2				2
	Pontal do Araguaia	*Panstrongylus geniculatus*						2		1	3
	Pontal do Araguaia	*Panstrongylus diasi*							1		1
	Ribeirãozinho	*Triatoma sordida*							1		1
		**Total**	**41**	**1**			**3**	**37**	**79**	**2**	**163**
**Total**			**41**	**1**			**3**	**54**	**129**	**7**	**235**

^A^ **N1:** Nymph stage 1; **N2:** Nymph stage 2; **N3:** Nymph stage 3; **N4:** Nymph stage 4. ^B^ ♂: Male. ^C^ ♀: Female. ^D^ NI: Natural infection.

Triatomine home invasions were reported in two municipalities: Pontal do Araguaia and Barra do Garças. Only one insect was found indoors at Pontal do Araguaia, which was captured in a bathroom and identified as *P. geniculatus*. In Barra do Garças, a total of 225 insects were captured in the urban areas, of which 64.4% were found in the intra-domicile area. Among these, the highest frequencies of capture occurred in bedrooms (35.6%), kitchens (12.4%), balconies (12.0%), and living rooms (8.0%). In the peridomicile, 19.1% of the insects were recorded. Considering entomological indicators, the colonization index for *T. williami* was 1.8%. Only one insect each was found at Pontal do Araguaia and Ribeirãozinho.

Captured insects were distributed across 13 neighborhoods in Barra do Garças, with a considerable number of insects in the Jardim Amazônia I, Jardim Pitaluga, and Jardim Mariano neighborhoods (Figure 2B). However, a hotspot in the heat map, caused by the identification of a triatomine colony, was observed only in the Pitaluga neighborhood (Figure 2B).

HUs with positive triatomine detection were located in neighborhoods close to the PESA Ecological Park. Our data suggested that household insect density decreased with increasing distance from the PESA to the HUs (p = 0.004).

### Natural infection and genotyping of Trypanosoma cruzi

In the parasitological examination, triatomines infected with *T. cruzi* were observed only in Barra do Garças, with an NI rate of 13.9%. Regarding cPCR analyses, significantly high NI rates were observed in two municipalities: Barra do Garças (65.7%) and Pontal Araguaia (25%). Among the adult triatomines, females had a higher infection rate than males (71.5% and 52.5%, respectively). Analysis of NI among triatomine species showed the highest rate in *T. williami* (76.2%), followed by *P. geniculatus* (50%) and *R. neglectus* (14.2%). Statistical analyses demonstrated that *T. williami* had a higher NI rate than *R. neglectus* (p = 0.001). The only infected specimen collected from Pontal do Araguaia was a female *P. geniculatus*.

**FIGURE 3:**
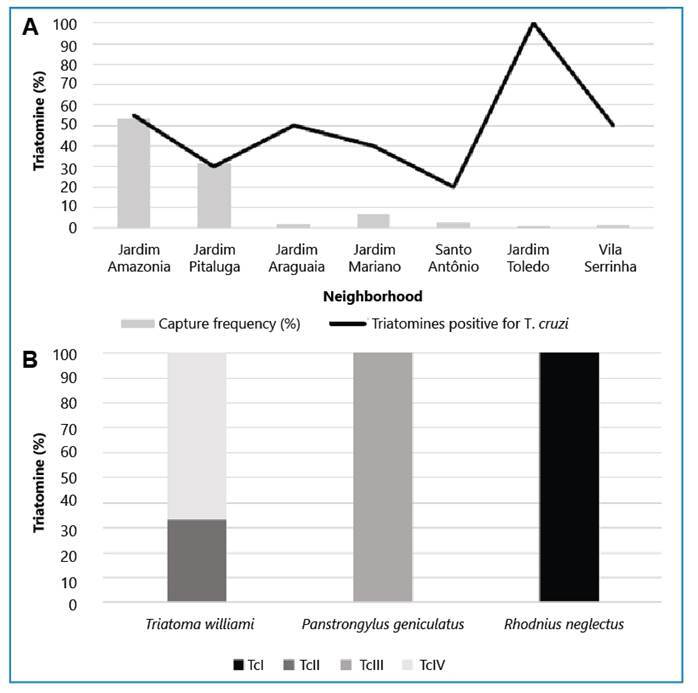
Natural infection and genotyping of *Trypanosoma cruzi*. **A.** Distribution of infected triatomines in the neighborhoods of Barra do Garças. **B.** Discrete typing units (DTUs) for *Trypanosoma cruzi* found in triatomine species. *T. cruzi* lineages: TcI, TcII, TcIII, and TcIV

The intradomestic and peridomestic environments had similar NI rates (64.9% and 66.6%, respectively). Triatomines infected with *T. cruzi* were detected in seven neighborhoods, with the highest number of infected insects collected in the Jardim Amazonia I and Jardim Pitaluga neighborhoods (Figure 3A).

Regarding the positive samples in the NI test, four DTUs were detected, including TcI, TcII, TcIII, and TcIV, in the triatomines collected from Barra do Garças. Regarding the only specimen analyzed from the municipality of Pontal do Araguaia, the presence of DTU TcIII was detected in *P. geniculatus*. Among the infected *T. williami* insects, TcII and TcIV were detected in specimens of both sexes, with TcIV identified in twice as many individuals as TcII (Figure 3B). Strains TcIII and TcI were detected in *P. geniculatus* and *R. neglectus* specimens, respectively (Figure 3B).

### Food sources of triatomines

Four food sources of triatomines were analyzed: birds, dogs, rodents, and humans. Dog DNA was not detected in any of the insects analyzed. Bird DNA was reported in one insect, and human DNA was reported in two insects in Pontal do Araguaia. Bird DNA was predominant in many insects (64.2%), followed by that of rodents (43.9%) and humans (32.8%) in Barra do Garças. Among the analyzed insects, DNA from more than one food source was detected in 62% of the samples. Among insects detected with only one food source, the frequency of finding birds as the food source was the highest (25%). Among insects with more than one food source, the frequency of birds/rodents was the highest (28.1%), followed by that of birds/rodents/humans (18.7%) (Table 2).

**TABLE 2:** Identification of the multiple food sources of triatomines collected from Barra do Garças.

Blood source	%
Birds	25
Rodents	6.2
Humans	6.2
Birds/Rodents	28.1
Birds/Humans	12.5
Rodents/Humans	6.2
Birds/Rodents/Humans	18.7

In two specimens of *R. neglectus,* birds were identified as food sources, and one was identified as feeding on humans. Two *P. geniculatus* specimens were observed to feed on birds, and one on rodents. *T. williami* was identified feeding on birds (64.8%), rodents (45.9%), and humans (37.8%). The feeding preference of *T. williami* for birds was confirmed by statistical analysis (p = 0.001). A difference in sex was observed when humans were considered the food source, with 41.3% for females and 18.1% for males. In total, 77% of the triatomines in which human DNA was detected showed NI to *T. cruzi*. Triatomines that fed on humans were not influenced by their location within the house where they were captured (p = 0.3) or by the sex of the specimen (p = 0.7).

## DISCUSSION

Socioeconomic conditions, rural-to-urban migration, urban growth, and environmental and climate change have contributed to the spread of ChD in the Americas^2^ ^,^ ^3^ ^,^ ^40^. In the ecological context of the disease, these factors facilitate the dispersal and adaptation of triatomine vectors to domestic environments^40^.

The five triatomine species observed in this study-*T. williami, T. sordida, P. geniculatus, P. diasi, and R. neglectus*-have previously been reported in the state of MT^24^ ^,^ ^26^ ^,^ ^28^ ^,^ ^41^ ^,^ ^42^ ^-^ ^49^. Among these species, *P. geniculatus, R. neglectu*s, and *T. sordida* have been classified as important in the epidemiology of ChD in Brazil because of their adaptation to the domestic environment and wide distribution throughout the country^3^ ^,^ ^21^. *T. sordida*, which typically exhibits low natural infection rates and primarily inhabits peridomestic ecotopes^3^ ^,^ ^21^, has recently been reported to colonize urban areas in São Paulo state^50^. In line with these findings, our study recorded the presence of *T. sordida* in peridomestic areas, and all specimens tested negative for *T. cruzi*.

*P. geniculatus* is widely distributed across South and Central America, with a confirmed presence in 19 Brazilian states and occurrence in all biomes under various climatic conditions^21^ ^,^ ^51^ ^,^ ^52^. Here, specimens of this species were collected from both intra- and peridomestic environments, reinforcing the synanthropic behavior previously reported in other studies^53^. *R. neglectus* is frequently associated with household invasions. It is primarily distributed in the states of Mato Grosso, Mato Grosso do Sul, Goiás, Minas Gerais, and Tocantins, with a strong presence in the Cerrado^48^ ^,^ ^54^ ^,^ ^55^. More recently, this species has been reported to colonize human dwellings in the states of Acre and São Paulo^56^ ^-^ ^58^. In the current study, most *R. neglectus* specimens were captured indoors, which is consistent with reports of home invasion in other regions^22^ ^,^ ^56^ ^-^ ^59^.

Previous studies have demonstrated the presence of *T. williami* in Barra do Garças^24^ ^-^ ^28^. Among all the specimens captured in the municipality, this species was the most predominant, with the majority of individuals collected being intradomicile. These findings highlight a concerning epidemiological scenario, as this triatomine species has previously been reported to invade domestic environments^24^ ^,^ ^25^ ^,^ ^59^. Moreover, the current detection suggests an elevated risk of vector-borne transmission among urban populations, particularly given the previous report of a colony discovered in the bed of a resident^28^. The establishment of this colony indicates the presence of both male and female insects in the environment, facilitating mating and potentially increasing female longevity^27^.Furthermore, this domiciliation may be combined with the adequate adaptation capacity of the vector^26^.

Barra do Garças has a distinct ecological context because of its proximity to the PESA Ecological Park. Such a context may facilitate contact between humans and sylvatic triatomines, which are potential vectors of ChD, and the human population^60^. A predominance of triatomines was observed in households near the PESA Ecological Park. This region of the Cerrado is characterized by spatial heterogeneity and high levels of plant and insect endemism^61^. Moreover, artificial lighting in the area contributes to the dispersal of these vectors toward human dwellings, as other studies have shown that the nocturnal flight of these insects is a challenge in preventing home invasion^62^ ^,^ ^63^.

Other alarming data were obtained from Barra do Garças, which showed a high NI rate for *T. cruzi* (65.7%). Previous research using cPCR reported an NI of approximately 30% in vectors belonging to the *T. williami* species^24^ ^,^ ^25^. In contrast, an NI rate of 39.4% was observed in triatomines using a parasitological diagnostic method, which is known to be less sensitive than molecular techniques^28^.

The species with the highest NI rate was *T. williami,* followed by *P. geniculatus* and *R. neglectus*. Reports of home invasion by these vectors are common, but with low levels of infection by *T. cruzi* ^39^ ^,^ ^64^ ^-^ ^66^. Once vectors infected with *T. cruzi* are identified within a household, the likelihood of ChD transmission to humans increases substantially.

Another important issue was the identification of the TcII and TcIV strains in *T. williami* specimens. The TcIV strain was detected in *T. williami* in a previous study^28^, which reported the highest incidence of this strain among insects captured in both the intra- and peridomiciliary environments.

The transmission cycle of DTU TcIV primarily occurs in sylvatic environments, with a distribution mainly in the Amazon region, and is associated with acute cases of ChD and oral transmission of the parasite^67^. DTU TcIV was first isolated from chronic human infections in Colombia^67^. TcII is predominantly associated with the domestic transmission cycle and is the primary cause of the severe cardiac forms of ChD in Brazil, often resulting in chronic cardiomyopathy and heart failure^67^ ^,^ ^68^. DTU TcII is linked to disease progression, including manifestations such as megaesophagus and megacolon^67^. 

In this context, investigating the blood meal sources of the vector is crucial because the *T. cruzi* genotype reflects the interaction between the vector and naturally infected reservoir hosts. This approach helps elucidate the dynamics of parasite transmission in both domestic and sylvatic environments^69^. The main reservoirs of the TcIV are carnivores, primates, and rodents^70^ ^-^ ^72^. In our study, insects infected with this strain tested positive for rodent and human DNA, but negative for canine DNA. These results support the hypothesis that rodents serve as potential reservoirs for DTU IV in the study region. 

DTU TcII was found in the analyzed triatomines, demonstrating the presence of rodent and human DNA in insects that were positive for DTU. TcIII and TcI were identified in *P. geniculatus* and *R. neglectus*, respectively, which carried bird and rodent DNA. Domestic animals can serve as reservoirs of *T. cruzi* in their home environments, contributing to the spread of ChD^73^ ^,^ ^74^. In the Brazilian states of Goiás and Distrito Federal, *P. geniculatus* and *R. neglectus* infected with *T. cruzi* have been found to invade homes and feed on human^75^ ^,^ ^76^.

Bird, human, and rodent DNAs were detected in *T. williami* specimens, with a statistically significant preference for birds. Corroborating our findings, a dietary preference for birds and dietary eclecticism in this species have been previously reported^77^. A recent study has shown that owls can serve as hosts for *T. cruzi* ^78^ *.* This raises the question of whether birds participate in the transmission cycle of the parasite beyond serving as food sources. A worrisome finding was that several *T. williami* specimens contained human DNA. To date, the use of human blood by this species has been observed only under experimental conditions^79^.

Although only seven cases of ChD have been officially reported in the state of Mato Grosso since 2007^80^, our findings suggest that these numbers may underestimate the actual epidemiological scenario, given the notably high detection of *T. cruzi*-infected triatomines that feed on human blood (37.8%). Rio Grande do Norte and Piauí showed a high frequency of vectors feeding on human blood, at rates of over 20% and 40%, respectively^81^ ^,^ ^82^. These data indicate that the situation observed in MT may occur silently in other regions of Brazil, potentially increasing the risk of vector-borne ChD transmission.

The findings of our study reveal the problem of intradomiciliary invasion by *T. cruzi*-infected triatomines in the urban areas of the study region in Mato Grosso. Additionally, our findings support the idea that ChD transmission by these vectors may pose a risk to the human population in Barra do Garças MT. Therefore, we recommend some preventive measures that may support surveillance efforts in the study area and other Brazilian regions with similar conditions: 1) active vector surveillance, particularly in regions with the highest occurrence of triatomines; 2) ongoing training for disease control agents and community health workers, especially those in direct contact with the population; 3) public education efforts should focus on improving community recognition of triatomine insects to enhance passive detection; and 4) health education programs in schools and higher education institutions, combined with outreach through electronic media, can effectively raise public awareness and promote preventive behaviors. Therefore, intensifying entomological surveillance is necessary to control and prevent ChD in the studied municipalities, particularly Barra do Garças.
