# Supplementary material for: Home invasion of triatomines (Hemiptera: Reduviidae) in the urban area of Mato Grosso, Brazil
Source: Rev Soc Bras Med Trop. 2025 Sep 22;58:e0119-2025. doi: 10.1590/0037-8682-0119-2025 (PMC12455752; doi:10.1590/0037-8682-0119-2025)
Supplement: Supplementary file 1 [file 1678-9849-rsbmt-58-e0119-2025-supp1.pdf]

SUPPLEMENTARY TABLE 01: Coordinates of the municipalities investigated in the middle Araguaia region, MT.

Municipality	Coordinate
Barro do Garças	15°53.24′ S, 052°15.24′ W, 318m
Campinápolis	14°32.35′ S, 052° 47.39′ W, 450 m
General Carneiro	15°42.39′ S, 052°45.19′ W, 343 m
Nova Xavantina	14°40.24′ S, 052°21.11′ W, 275 m
Novo São Joaquim	14°54.21′ S, 053°01.06′ W, 400 m
Pontal do Araguaia	15°50.43′ S, 052°00.33′ W, 370 m
Ponte Branca	16°45.51′ S, 052°50.00′ W, 424m
Ribeirãozinho	16°29.07′ S, 052°41.32′ W, 477 m
Torixoréu	16°11.58′ S, 052°33.20′ W, 335 m
