# Supplementary material for: Home invasion of triatomines (Hemiptera: Reduviidae) in the urban area of Mato Grosso, Brazil
Source: Rev Soc Bras Med Trop. 2025 Sep 22;58:e0119-2025. doi: 10.1590/0037-8682-0119-2025 (PMC12455752; doi:10.1590/0037-8682-0119-2025)
Supplement: Supplementary file 2 [file 1678-9849-rsbmt-58-e0119-2025-supp2.pdf]

SUPPLEMENTARY TABLE 02: Description of reagent concentrations used in cPCR for detecting natural infection (NI), genotyping *T. cruzi,* and identifying triatomine food sources.

cPCR	Primer	Reagents used in the reaction	Electrophoresis	Reference
T. cruzi NI	P21	1× PCR buffer was used (50 mM Tris-HCl [pH 8.0], 100 mM NaCl, 0.1 mM EDTA, 5 mM DTT, 50% glycerol, and 1.0% Triton®X-100), 1.5 mM $MgCl_2$. 1 U Taq High Fidelity (Cellco), and 25 ng of DNA from each sample.	2% agarose gel stained with 0.5 µg/mL ethidium bromide	Adapted from Brígido *et al.* (2017).
Genotyping of *T. cruzi*	*TcSC5D*	1X PCR buffer (Tris-HCl pH8, KCl), 2 mM $MgCl_2$, 10 pMol of primers TcSC5D-forward 5′-GGACGTGGCGTTTGATTTAT-3′ and TcSC5D-reverse 5′-TCCCATCTTCTTCGTTGACT-3′, 200 µM dNTP, 2.5 U Taq High Fidelity (Cellco), and 25 ng of DNA from each sample.	1.2% agarose gel stained with 0.5 µg/mL ethidium bromide	Cosentino and Agüero, 2012
Triatomine Food Sources	Avian, *Canis familiaris, Homo sapiens, Rodent* (Table 03)	50 mM Tris-HCl (pH 8.0), 1.5 mM $MgCl_2$, 200 nM Fw/Rv primer, 0.2 mM dNTP, and 1U Taq High Fidelity (Cellco), 1.5 mM MgCl2, and 100 ng of DNA for a final volume of 25 µL were used in cPCR reactions.	1.5% agarose gel stained with 0,5 µg/ mL ethidium bromide	Walker et al., 2004; Ribeiro Jr. et al., 2015

cPCR: conventional conventional Polymerase Chain Reaction; **NI:** Natural Infection; **mM:** millimolar; **µM:** Micromolar; **µg:** Micrograms; **$MgCl_2$:** Magnesium chloride; **ng:** nanograms; **µL:** Microliters; **HCL:** Hydrochloric acid; **EDTA:** Ethylenediaminetetra acetic acid; **DTT:** Dithiothreitol; **dNTP:** Deoxyribonucleotide triphosphate; **KCL:** Potassium Chloride; **pM:** Picomole; **nM:** nanomole
