# Supplementary material for: Home invasion of triatomines (Hemiptera: Reduviidae) in the urban area of Mato Grosso, Brazil
Source: Rev Soc Bras Med Trop. 2025 Sep 22;58:e0119-2025. doi: 10.1590/0037-8682-0119-2025 (PMC12455752; doi:10.1590/0037-8682-0119-2025)
Supplement: Supplementary file 3 [file 1678-9849-rsbmt-58-e0119-2025-supp3.pdf]

SUPPLEMENTARY TABLE 03: List of food sources analyzed for triatomine gut DNA identification, including primer sequences, amplicon size (bp), cPCR annealing temperature, and corresponding references.

Name	*Primer* (5'-3')	Size (bp)	°C	Reference
Avian	R – ATAGAATGGCCTGGGTTGAAAAG<br>F – AAGTTTTTCACACAGAGGGTGGT	197	60	Walker *et al.*, 2004
Canis familiaris	R – GTCAATGGTTTCAGGACATATAGTTTT<br>F – TATTGTATGCACTTAGTCCTGTTTTTG	476	58	Ribeiro jr.*et al.*, 2015
Homo sapiens	R – GTAGTACATAAAAACCCAATCCACATC<br>R – GTCGGATACAGTTCACTTTAGCTACC	346	58	Ribeiro jr.*et al.*, 2015
Rodent	R – CAAGACGGATGATCAAAATGTG<br>F – ATTGGGTGGCTGTATATGTATGG	161	57	Walker *et al.*, 2004

bp: base pairs; **°C:** degrees Celsius; **R**: Reverse; **F:** Forward.
